# Supplementary material for: Multimodal imaging and functional analysis of the chick NMDA retinal damage model
Source: PLoS One. 2021 Sep 7;16(9):e0257148. doi: 10.1371/journal.pone.0257148 (PMC8423281; doi:10.1371/journal.pone.0257148)
Supplement: S1 File — (ZIP) [file pone.0257148.s004.zip › SD-OCT/C200727/010/D8-PWk2/C200727-010_OD_V_14X14_0_REGAVG0000042/Manual_PreDefineFiveByFiveGridRectangularRetina_1.pdf]

# Five By Five Grid Template Report

| | | | |
|---|---|---|---|
| **Patient ID** | C200727 | **Name** | See InVivoVue |
| **Exam Date** | See InVivoVue | **Exam Time** | See InVivoVue |
| **Eye** | OD | **Averaged** | N/A |
| **Depth (mm)** | 1.65 | **Depth Samples** | 1024 |
| **Pitch (um/pix)** | 35.000 x 35.000 x 1.608 | **Refractive Index** | See InVivoVue |
| **Scan Type** | Rectangular 14.000mm x 14.000mm x 1.647mm ,<br>400 x 400 x 1024 | | |

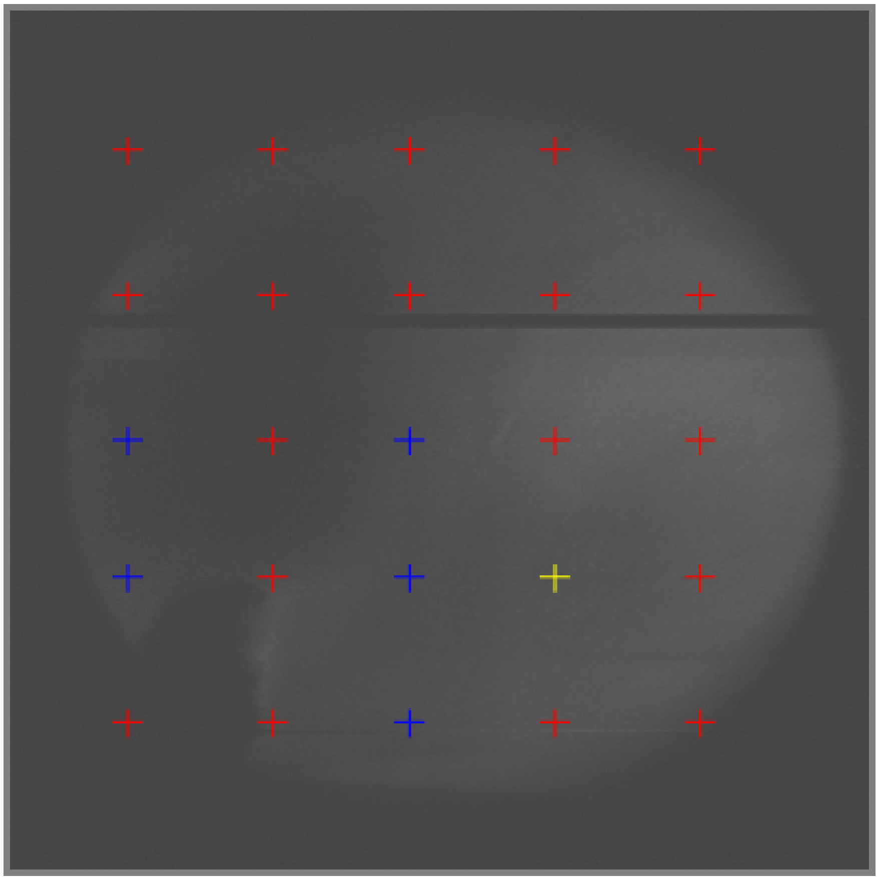

## Thickness Metrics

| Analysis | Average(mm) | Minimum(mm) | Maximum(mm) | STDEV(mm) |
|---|---|---|---|---|
| **IRNFL - RPE** | 0.2802 | 0.2671 | 0.2933 | 0.0110 |
| **IRNFL - ORNFL** | 0.0444 | 0.0337 | 0.0637 | 0.0122 |
| **ORNFL - OPL** | 0.1558 | 0.1398 | 0.1729 | 0.0133 |
| **OPL - ETPRS** | 0.0603 | 0.0555 | 0.0674 | 0.0044 |
